# South Asian American Perspectives on Overweight, Obesity, and the Relationship Between Weight and Health

**DOI:** 10.5888/pcd9.110284

**Published:** 2012-05-31

**Authors:** Joyce W. Tang, Maryann Mason, Robert F. Kushner, Manasi A. Tirodkar, Neerja Khurana, Namratha R. Kandula

**Affiliations:** Author Affiliations: Maryann Mason, Robert F. Kushner, Neerja Khurana, Namratha R. Kandula, Feinberg School of Medicine, Northwestern University, Chicago, Illinois; Manasi A. Tirodkar, National Committee for Quality Assurance, Washington, DC.

## Abstract

**Introduction:**

Compared with other racial groups, South Asian adults develop type 2 diabetes and cardiovascular disease at a lower body mass index (BMI). Perceptions of weight and the effect of weight on health can influence weight-loss attempts but are not well described in this population. The objective of this study was to examine perceptions of weight appropriateness and the effect of weight on health among South Asian Americans.

**Methods:**

We recruited 75 South Asian American adults from a single metropolitan area in the Midwestern United States. During individual, face-to-face interviews, we asked participants what they think about their weight and how weight affects their health. We measured their weight and height and calculated BMI. Each interview was audiotaped, transcribed verbatim, and translated into English. We conducted analyses using NVivo software. A second investigator coded 20% of interviews to verify coding consensus.

**Results:**

Sixty-seven percent of participants were overweight or obese; 40% of overweight participants and 12% of obese participants perceived themselves to be normal weight or underweight. Forty-eight percent of overweight and 82% of obese participants believed their weight affected their health. Participants commonly cited physical problems as being associated with their weight, but few connected their weight with risk for chronic diseases.

**Conclusion:**

South Asian Americans may underestimate their weight status and the effect of their weight on their risk for chronic diseases. Interventions to promote weight loss among South Asian Americans should focus on modifying perceptions of normal weight and personalizing the relationship between overweight and chronic diseases.

## Introduction

Almost 3 million South Asians (Asian Indian and Pakistani) live in America; during the past decade they have been the second-fastest growing racial/ethnic group in the United States (1). Among all Asians in the United States, South Asians have the highest rates of overweight/obesity (25% among men and 37% among women) (2,3). South Asians develop insulin resistance at a lower body mass index (BMI [kg/m^2^]) than do other racial/ethnic groups; as a result, they have a higher prevalence of type 2 diabetes and cardiovascular disease (4-6). In response to the increased risk for comorbid conditions in Asian populations, the World Health Organization (WHO) in 2002 recommended establishing new BMI standards for Asian populations: normal weight (18.5 to <23.0 kg/m^2^), a moderate-risk public health action point (23.0 kg/m^2^), and a high-risk public health action point (27.5 kg/m^2^) (7). In 2009, India’s health ministry adopted even lower BMI ranges for overweight (23.0 to <25.0) and obesity (≥25.0) (8).

As average BMI has increased in all populations, misperceptions about one’s weight status have become more common (9,10). These misperceptions could impede weight-loss efforts because accurate self-perception of overweight and perceived personal health risk are necessary precursors to weight-loss attempts (11). Among South Asian Americans, who are more susceptible to the deleterious effects of weight gain than most other racial/ethnic groups (4-6), little is known about self-perceptions of weight status and weight-related health risks. The primary objective of this study was to examine perceptions of weight appropriateness and the effect of weight on health among English-, Hindi-, and Urdu-speaking South Asian Americans. A secondary objective was to explore the role of age, sex, education level, length of stay in United States, and preferred language in such perceptions.

## Methods

### Study setting and participants

This qualitative study was part of a larger study on the concepts of health and disease, with a particular focus on cardiovascular disease, among South Asian Americans. The parent study was approved by the Northwestern University Feinberg School of Medicine institutional review board. We conducted semistructured interviews of South Asian men and women recruited between December 11, 2006, and May 24, 2007. We recruited participants from a federally qualified health center and a community center that provides immigrant services not related to health care (eg, English language and citizenship classes). Both centers are located on the north side of Chicago, where 30% of the Chicago metropolitan area’s South Asians reside. The population served by the health center is low income; 80% of its patients have an income below 100% of the federal poverty level. Compared with the general South Asian population in the United States, the neighborhood served by both centers includes a higher percentage of recent immigrants who have limited English proficiency and higher rates of poverty (12).

Of 168 people approached for participation, 77 (46%) agreed to be interviewed. Inclusion criteria were being aged 20 through 75, self-identifying as Asian Indian or Pakistani, and speaking English, Hindi, or Urdu. We made an effort to recruit an equal number of men and women among all age groups and among the 3 languages. We compared the sociodemographic characteristics of our participants to those of the Devon Avenue South Asian community, as described in the 2000 Census, and they were similar in age, language, country of origin, and years in the United States (12).

The project coordinator or staff members at the health or community center approached and recruited most participants. Some participants notified family or friends about the study, and these people were also included if they met inclusion criteria. No participants were excluded from participation in the interviews; however, during analysis, we excluded 2 participants because of concerns that their cognitive deficits rendered their responses unreliable; our final sample included 75 participants, 48 from the health center and 27 from the community center. We gave each participant $20 for participation.

### Interviews

This study focused on portions of the interview that relate to perceptions of weight and the relationship between weight and health. Through semistructured interviews conducted at the health or community center, a single project coordinator (fluent in English, Hindi, and Urdu) asked participants what they think about their weight, whether they think their weight affects their health, and, if they think their weight affects their health, how? At the end of the interview, the project coordinator collected data on participants’ sex, age, education, religion, length of stay in the United States, and insurance coverage. The project coordinator also measured weight (to the nearest 0.5 lb) and height (to the nearest 0.1 cm). At the health center, we used a calibrated Seca 700 balance-beam scale with measuring rod (Seca, Hamburg, Germany); at the community center, we used a Taylor LED digital scale (Taylor Precision Products, Oak Brook, Illinois) and a flexible tape measure taped to the wall. We calculated BMI for all participants. All interviews for the parent study were audiotaped, transcribed verbatim, and translated into English; each lasted between 30 and 45 minutes.

### Coding scheme

We used 5 interviews to create a comprehensive coding guide. We developed an initial coding scheme, which was modified as needed during the coding process. Themes included 1) perceived weight status, 2) method used to assess weight status, 3) presence or absence of perceived personal health risk related to one’s weight, 4) ways in which one’s personal health is affected by one’s weight, and 5) general health risks an individual could experience because of overweight (eg, if participants suggested that being overweight is a risk factor for a heart attack). Using the following guidelines, we coded perceived weight status as underweight if participants described their weight as low or needing to gain; normal weight if they described their weight as normal or fine; or overweight if they described their weight as high, overweight, or needing to lose. For the analysis on perceived weight status, we excluded 18 participants whose response to this question did not fit any of the 3 categories (eg, described weight as increasing, decreasing, or maintained). We did not differentiate between perceived overweight and perceived obesity. Patients were not asked explicitly about the method they used to assess their weight status, but for participants volunteering this information, we identified 3 major categories of responses: 1) alignment with height or BMI; 2) alignment with physician opinion; and 3) alignment with age. We coded perceived personal health effects related to one’s weight as present or absent; we excluded 11 participants whose response to this question was ambiguous (ie, discussed general effects of weight or weight gain but did not comment about effect on self). We categorized weight-related health problems as physical (eg, difficulty walking) or related to chronic disease (eg, hypertension).

We used 2 BMI classification schemes for actual weight status. In our main analysis, we used classifications recommended by WHO and National Institutes of Health for the general population: underweight (<18.5), normal weight (18.5 to <25.0), overweight (25.0 to <30.0), and obese (≥30.0) (13). We also conducted analyses using WHO-defined BMI categories for public health action in Asians: normal weight (18.5 to <23.0), moderate risk or overweight (23.0 to <27.5), and high risk or obese (≥27.5) (7,14).

### Data analyses

The first author (J.W.T.) coded all 75 interviews using NVivo software (QSR International Pty Ltd, Doncaster, Victoria, Australia). A second investigator (M.M.) coded 20% of the interviews to verify coding consensus and establish intercoder reliability. Initial intercoder reliability was 83%; it increased to 100% after discussion of areas of disagreement. We used descriptive statistics to describe the proportions of participants who were overweight or obese, whose perceived weight status was discordant with actual weight status, and who perceived their weight to affect their personal health. We used the Fisher exact test to assess differences in 1) weight status by sociodemographic characteristics and 2) underestimation of weight status or the effect of weight on health by sociodemographic characteristics. Statistical significance was defined as *P* < .05. In these analyses, we categorized actual weight status by using WHO BMI classifications for the general population. Sociodemographic variables included were age, sex, education, length of stay in United States, and language preferred for the interview. Although this study was not powered to detect differences by sociodemographic characteristic, we conducted these analyses to explore possible hypotheses. We also identified interview responses that illustrate important themes.

## Results

### Participant characteristics

Fifty-one percent of participants were men (Table 1). The mean age of participants was 46 years (standard deviation, 15.2 y). Equal numbers of participants aged 20 to 39 and 40 to 59 participated. Despite efforts to recruit more people aged 60 or older, we recruited only 17. Twenty-one percent (n = 16) did not have a high school diploma; 65% had attended at least 1 year of college. Fifty-five percent of participants were uninsured. Sixty-five percent of participants had been in the United States for 10 years or less, and 69% preferred their interviews in Hindi or Urdu.

**Table 1:** Characteristics of Participants (N = 75) in Qualitative Study on Overweight and Health Among South Asians, Chicago, Illinois, 2006–2007

Characteristic	n (%)^a^
**Sex**	
Male	38 (51)
Female	37 (49)
**Age, y**	
20–39	29 (39)
40–59	29 (39)
≥60	17 (23)
**Education**	
No high school diploma	16 (21)
High school diploma	10 (13)
At least 1 year of college	49 (65)
**Insurance**	
None	41 (55)
Public aid	18 (24)
Private insurance	9 (12)
Do not know^b^	7 (9)
**Preferred language for interview**	
Urdu	28 (37)
Hindi	24 (32)
English	23 (31)
**Length of stay in United States, y**	
≤10	50 (67)
>10	25 (33)
**Religion**	
Muslim	51 (68)
Hindu	16 (21)
Other^c^	8 (11)
**WHO-defined BMI categories for the general population, kg/m^2^**	
Underweight (<18.5) or normal weight (18.5 to <25.0)	25 (33)
Overweight (25.0 to <30.0)	30 (40)
Obese (≥30.0)	20 (27)
**WHO-defined BMI categories for Asian populations, kg/m^2^**	
Underweight (<18.5) or normal weight (18.5 to <23.0)	11 (15)
Overweight (23.0 to <27.5)	29 (39)
Obese (≥27.5)	35 (47)

Abbreviations: BMI, body mass index; WHO, World Health Organization.

^a^ Percentages may not sum to 100% because of rounding.

^b^ Includes those who did not know whether they had insurance or those who had an insurance card but did not know what it was for.

^c^ Other religions were Sikh and Christian.

According to general-population BMI categories, 40% of participants were overweight and 27% were obese. Participants aged 40 or older were more likely than younger participants to be overweight or obese (76% vs 53%; *P* = .05). We found no differences in the proportion of overweight or obese participants by sex, education, length of stay in United States, or preferred language.

### Weight perception and method for assessing weight appropriateness

Forty percent of overweight participants and 12% of obese participants perceived themselves to be normal weight or underweight (Table 2). One participant commented that his weight had increased because “I build up a lot of muscle in my body, so there is the reason I gain weight but it is not bad” (man, aged 46, overweight). Another participant stated, “The doctor advised me to reduce 10 pounds of weight, but I have no problem with my weight and I don’t consider myself as obese” (woman, aged 47, overweight). For assessing weight appropriateness, 12 participants referenced BMI or their weight being in accordance with their height, 6 mentioned their physician’s opinion, and 5 mentioned their weight in accordance with their age. Overweight or obese participants who were aged 40 or older were more likely than younger overweight or obese participants to perceive their weight as normal, although this difference was not significant (36% vs 8%; *P* = .12). Perception of one’s weight status did not differ by sex, education, length of stay in the United States, or preferred language.

**Table 2:** Perceived Weight Status and Perceived Health Effect Among Participants (N = 75) by Weight Category, Qualitative Study on Overweight and Health Among South Asians, Chicago, Illinois, 2006–2007

BMI Category	Actual Weight Status, n (N = 75)	Actual Weight Status of Participants in Analysis of Self-Perceived Weight, n (n = 57)^a^	Perceives Self to Be Normal Weight or Underweight^b^, n (n = 57)^a^	Actual Weight Status of Participants in Analysis of Self-Perceived Health Effects, n (n = 64)^c^	Perceives Personal Health Effects Due to Weight^d^, n (%) (n = 64)^c^
**WHO-defined BMI categories for the general population (kg/m^2^)**					
<18.5 (Underweight) or 18.5 to <25 (normal weight)	25	20	17 (85)	20	NA
Overweight (BMI 25.0 to <30.0)	30	20	8 (40)	27	13 (48)
Obese (BMI ≥30.0)	20	17	2 (12)	17	14 (82)
**WHO-defined BMI categories for Asian populations (kg/m^2^)**					
<18.5 (Underweight) or 18.5 to <23 (normal weight)	11	10	10 (100)	9	NA
Overweight (BMI 23.0 to <27.5)	29	20	12 (60)	24	5 (21)
Obese (BMI ≥27.5)	35	27	5 (19)	31	23 (74)

Abbreviations: BMI, body mass index; WHO, World Health Organization; NA, not applicable to analysis.

^a^ Analysis excludes 18 participants whose perceptions could not be categorized.

^b^ Perceived weight status was coded as underweight if participants described their weight as low or needing to gain; normal weight if they described their weight as normal or fine; and overweight if they described their weight as high, overweight, or needing to lose.

^c^ Analysis excludes 11 participants whose responses could not be categorized.

^d^ Perceived health effects of weight were coded as present or absent.

### Weight-related risk perception and perceived effect of weight on health

Forty-eight percent of overweight and 82% of obese participants perceived their personal health to be affected by their weight (Table 2). Such perceptions did not differ by age, sex, education, length of stay in the United States, or preferred language. Overweight or obese participants who perceived their weight not to affect their personal health often explained that their weight did not impose physical limitations. For example, 1 participant stated, “No, [my weight is] not affecting my health, I mean, it’s keeping it well, because if my weight would be high then I will have trouble walking or climbing stairs” (woman, aged 57, overweight). Among those who felt their weight affected their personal health, 15 participants described physical problems (eg, shortness of breath, joint aches, lethargy, decreased ability to exercise), and 3 made a connection to chronic diseases (eg, high blood pressure, high cholesterol, diabetes). Although few overweight and obese participants associated their own weight with an increasing risk for developing chronic diseases, 40% of participants suggested a general connection between overweight and development of chronic diseases. We identified 3 domains on the perceived effect of weight on personal and general health (Table 3).

**Table 3:** Representative Comments on Perceptions of the Personal and General Effects of Weight on Health, Qualitative Study on Overweight and Health Among South Asians, Chicago, Illinois, 2006–2007

Domain	Comment
Physical problem related to one’s own weight	“I think my lower back pain is because of that [my weight]. I can’t stand for long time because ankle and feet is bearing the entire weight” (woman, aged 29 years, overweight^a^).
	“I feel dullness because of weight, I feel tiredness in my movement, a little activeness what they call it is not there” (man, aged 57, obese^a^).
	“Well when you gain more weight, you get more lazy, you get more sleepy, you get tired early, like you have to you know carry the whole weight with you all the time, it makes you tired” (man, aged 26, overweight^a^).

Chronic disease problem related to one’s own weight	“I never had any problem ‘til now, but it has started happening now because my weight has increased. I could get diabetes . . . [my doctor] said that I am borderline . . . If my *weight^b^* keeps on increasing like this and if *cholesterol* keeps on increasing then *heart attack* will surely happen” (man, aged 33, obese^a^).
	“I did have the cholesterol last year and . . . I figure out that doctor want to put me on . . . medicines and I didn’t took medicines so I did exercise lose my weight so I get better so after that it was fine but the triglycerides go up a little bit here and there if I watch myself I go okay” (man, aged 44, overweight^a^).

Chronic disease problems related to weight in general	“[My father had a heart attack because] he smoked a lot, he was very smart and active body, but . . . he was kind of fat” (woman, aged 60, overweight^a^).
	“Yeah, because if your *weight* is in excess then obviously it will cause harm to you from inside. If grease increases then it affects your *heart”* (woman, aged 39, overweight^a^).

^a^ The weight category listed refers to body mass index categories defined by the World Health Organization for the general population.

^b^ Italics denote words for which a participant switched to a different language during the interview.

### Analyses using Asian-specific BMI categorization

According to Asian-specific BMI categories, 39% of participants were overweight, and 47% of participants were obese (Table 1). Sixty percent of overweight participants and 19% of obese participants perceived themselves to be normal weight or underweight (Table 2). Twenty-one percent of overweight participants and 74% of obese participants perceived their health to be affected by their weight. Compared with participants classified as overweight according to general population categories, participants classified as overweight by Asian-specific BMI categories were more likely to perceive themselves to be normal weight and less likely to perceive their health to be affected by their weight. We observed no differences between obese participants classified by general population BMI and Asian-specific BMI categories.

## Discussion

In our study of South Asian Americans, the prevalence of overweight and obesity were high, and underestimation of weight status and the consequences of being overweight were common. Misperceptions about weight status and weight-related health consequences were particularly common among participants who had a BMI between 23 and 27.5; these people are considered overweight or moderate risk according to Asian-specific categories, but about half are considered normal weight according to general population categories. To our knowledge, ours is the first research study describing perceptions of weight and the health effects of weight in South Asian Americans, a population at high risk for diabetes and cardiovascular disease (15,16).

Our finding that South Asian Americans underestimate their weight status and weight-related health consequences is not surprising. These underestimations are common and increasingly prevalent among the general population of the United States (9). Among white populations, approximately one-third of overweight and 5% of obese people perceive their weight to be normal (10). Among Hispanic and African American populations, the magnitude of underestimation is even higher (10,17-19). Studies in the United Kingdom indicate that overweight South Asian women are more likely than white women to underestimate their weight status (20,21) and to equate a larger body size with health (22). Although our study did not compare South Asians with other racial/ethnic groups, the degree of underestimation of weight status may be greater among South Asians than among other groups.

Although study participants frequently cited physical weight-related problems, few associated their weight with the risk for developing chronic diseases. Many participants were aware in general that excess weight can contribute to chronic diseases, but most did not personalize this risk. The gap between general risk perception and personal risk perception has been documented in other medical conditions, including HIV (23). Lack of a perceived connection between one’s weight and chronic disease risk has been reported among other populations (24,25), but it may be a particular problem for South Asian Americans, who develop insulin resistance (and thus diabetes and cardiovascular disease) at a lower BMI than do other racial/ethnic groups.

Our study suggests a possible association between increasing age and greater underestimation of weight status, which deserves further exploration in future studies. Increasing age has been positively correlated with misperception of weight status in other studies and populations, including adults in Pakistan (26). These misperceptions may relate to acceptance of weight gain with age, greater prevalence of overweight among older people, and the tendency for people to assess their weight status in comparison with their peers. Studies among white populations have suggested that overweight women are more likely than men to agree that they are overweight (27), but our study did not identify any differences by sex.

Our study has several strengths. First, the qualitative research design allowed for a richness of data not found in secondary data analyses. Second, half the participants were men, for whom data are lacking in research on weight. Third, this research addresses weight perceptions among a low-income and underinsured South Asian population that prefers to communicate in a non-English language — a population that may be harder to reach and at even higher risk for diabetes and cardiovascular disease than the general South Asian population (28,29). Fourth, BMI was calculated from measured heights and weights, not self-reported data. Finally, we used rigorous qualitative methods; 20% of the surveys were coded by 2 investigators.

Our study has several limitations. We used a convenience sample from a federally qualified health center and a community center in a single metropolitan area; the sample size was not sufficient to evaluate differences between Indian and Pakistani Americans. Although our participants were similar demographically to the South Asian population of the neighborhood from which the sample was drawn, our results may not be generalizable to all South Asians in the United States (12). Second, open-ended responses related to perceived weight status were not corroborated with close-ended categorical classification. Third, because participants were not probed about the many ways in which their weight could affect their health, their responses reflect a prioritized, rather than comprehensive, view. Fourth, because our study was not powered to evaluate differences by sociodemographic characteristic, our analyses should be considered exploratory. Fifth, our data were collected several years ago, but they are still relevant because societal attitudes toward weight have not changed much since then. Despite aggressive public health campaigns to increase awareness and understanding of the concept of healthy weight, the general population continues to under-recognize overweight and obesity (30).

Our study suggests that interventions to promote weight loss among South Asian Americans should focus on reforming perceptions of normal weight, establishing the connection between overweight and the development of chronic diseases, and strengthening the perceptions of personal risk. These measures will be difficult to achieve unless the US health care system formally adopts lower BMI cut-offs for overweight and obesity for South Asian Americans.
